# Geolocator Tracking and Stable Isotope Analysis Suggest Mixed Migration Strategies in White‐Shouldered Starlings (*Sturnia sinensis*)

**DOI:** 10.1002/ece3.71151

**Published:** 2025-03-30

**Authors:** Caroline Dingle, John A. Allcock, Pia M. C. Ricca, Paul J. Leader, Chloe E. R. Hatten, Juha Merilä

**Affiliations:** ^1^ Area of Ecology and Biodiversity, School of Biological Sciences The University of Hong Kong Hong Kong Hong Kong SAR; ^2^ Biology Department Capilano University North Vancouver British Columbia Canada; ^3^ Aurecon, Commercial Centre Yuen Long New Territories Hong Kong SAR; ^4^ Ecological Genetics Research Unit, Research Program in Organismal and Evolutionary Biology, Faculty Biological and Environmental Sciences University of Helsinki Helsinki Finland

**Keywords:** East Asian–Australasian Flyway, geolocation, landbirds, migration, stable isotopes

## Abstract

The East Asian–Australasian Flyway (EAAF) is one of the least studied flyways, especially for migratory passerines. A better understanding of the migratory behaviour of birds along the EAAF is critical to inform conservation efforts and to improve the understanding of the evolution of migratory behaviour. White‐shouldered starlings ( *Sturnia sinensis* ) breed in southern China and northern Vietnam, with at least some populations migrating to distinct wintering grounds. Using a combination of extrinsic (geolocators) and intrinsic markers (stable isotopes), we tested whether a presumed resident breeding population in Hong Kong is resident or migratory. We discovered that most tagged individuals (7/8) migrated southwest from Hong Kong to winter in Vietnam/Cambodia up to 2013 km from their breeding grounds. We also found evidence of an alternative migration pathway, with one individual migrating northeast to overwinter in southern China. Stable isotope analyses of feathers revealed that the northeast‐migrating individual had different δ^2^H and δ^34^S values than those migrating southeast in that year (but had similar isotope values in the previous year). Based on isotope data alone, a second individual was identified who had distinct δ^2^H and δ^34^S values between years, suggesting that this individual also switched wintering grounds between the two breeding seasons. Together our results confirm that the Hong Kong breeding population is migratory, and both geolocator and stable isotope data suggest that the Hong Kong population of this species might host at least two different migration strategies, with some individuals switching their wintering grounds between years.

## Graphical abstract

Using both intrinsic and extrinsic markers, we revealed multiple migration strategies for white‐shouldered starlings breeding in Hong Kong. Birds from this site migrated to multiple wintering sites, and some birds appeared to switch wintering sites between years. Our results provide further insights into the movement of passerines along the East Asian–Australasian Flyway.

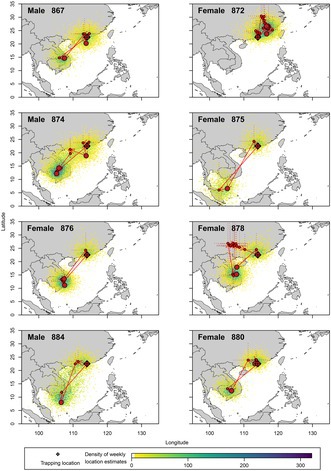

## 1 Introduction

Every year, millions of birds undertake annual migrations—regular movements between breeding and wintering grounds (Berthold 1999; Kirby et al. 2008). Because of geographic and habitat constraints, multiple species occupying the same geographic region often follow similar migratory routes, or ‘flyways’, between breeding and non‐breeding sites (Kirby et al. 2008). Of the eight global flyways, the East Asian–Australasian Flyway (EAAF) supports the highest diversity of birds, including the highest number of threatened species (Yong et al. 2015) yet it is one of the least well studied (Yong et al. 2021; McKinnon and Love 2018). Current information on this flyway is skewed towards waterbirds and birds of prey (Yong et al. 2015; Bridge et al. 2014); terrestrial songbirds along this flyway are less well studied, although many species are at great risk during their migration due to factors including hunting (for food or caged bird trade), habitat destruction, and climate change (Kirby et al. 2008; Yong et al. 2015). Gaining a better understanding of the migratory behaviour of terrestrial songbirds throughout this geographic region, including the use of different habitats for breeding, stopover, and wintering sites, can inform evolutionary studies and conservation management for this region.

Migratory movements of large birds are commonly tracked using extrinsic markers such as satellite or GPS tags, attached to the legs, wings, or backs of migrating individuals. While these methods have the advantage of high spatial accuracy, most of these tags are still too heavy to attach to small passerines (Bridge et al. 2013). Light‐level geolocators, which determine position based on geographical differences in daylength, have the advantage of being light enough to be used on smaller birds (Bridge et al. 2013; McKinnon and Love 2018). The trade‐off is that they come with large error margins, particularly in the north–south direction (100s of kms rather than 10s; Fudickar et al. 2012). Additionally, geolocators need to be retrieved from the bird in order to download the data (rather than being able to download the data remotely). While geolocators have been widely applied across the Atlantic flyways, as of 2018 less than 1% of all studies using geolocators to track migration movements were conducted along the EAAF (McKinnon and Love 2018). In the past few years, studies using geolocators have provided data on migration in a growing number of passerines using the EAAF, particularly birds breeding in Russia or Japan (Koike et al. 2016; Yamaura et al. 2017; Heim et al. 2018, 2020, 2022, 2023, 2024; Adams et al. 2022; Bensch et al. 2022; Turbek et al. 2022; Tian et al. 2024; Zhao et al. 2024). These studies have mostly focused on species with relatively long migrations, breeding in temperate regions (particularly Japan and Eastern Russia) and migrating to the tropics (Yong et al. 2021). Less is known about the migration behaviour of birds breeding in Southern China, where conditions can be suitable to support year‐round residency (but see Tian et al. 2024).

Pairing geolocator data with data from an intrinsic marker can help overcome some of the drawbacks of this method by providing additional data to corroborate estimates of wintering and breeding grounds. Stable isotope analysis (SIA) is one technique that has been used to provide information on migratory movements and to connect breeding and wintering grounds (Rundel et al. 2013; Hobson and Wassenaar 2019; Szarmach et al. 2024). SIA has been applied to address questions about migratory behaviour and connectivity for passerines using other flyways, including birds using Asian breeding grounds (e.g., Jong et al. 2019). While a growing number of studies have applied SIA to study migration along the EAAF (e.g., Weng et al. 2014; Choi et al. 2020), the utility of this method for tracking migration in Asia remains largely untested. In a study comparing migration data collected from satellite tags and stable isotope data (δ^2^H) on migratory ducks, stable isotope data did not correlate well with estimated moult locations derived from satellite data but did show similar patterns in the degree of variation in migratory behaviour (Bridge et al. 2014). These results suggest that the combination of extrinsic tracking data with stable isotope data can provide information about migratory behaviour.

Several different isotopes can be used in studies of animal migration. Stable isotope ratios of hydrogen (δ^2^H) have been most commonly used to track animal migration due to the broad spatially predictable geographic patterns of isotopic variation resulting from differences in rainfall patterns, which can be visualised in an isoscape (Hobson 1999; Bridge et al. 2014; Hobson and Wassenaar 2019). Hydrogen consumed by birds through their diet becomes incorporated in their tissues, thus providing a signal of the geographic locality where that tissue was grown (Hobson and Wassenaar 2019). Oxygen isotopes are sometimes included as an additional parameter for migratory studies and are predicted to show similar patterns of variation to hydrogen, but oxygen isotope ratios are more difficult to interpret in studies of animal migration due to differences in how both elements are incorporated into tissues (Hobson and Wassenaar 2019; Choi et al. 2020). Other isotopes, such as those of carbon, nitrogen, and sulphur, also show variation between sites, largely due to dietary differences, but this variation is not as spatially predictable over large scales (Wassenaar 2008; Hobson and Wassenaar 2019). Variation in δ^34^S has been shown to vary between birds using coastal versus inland habitats (Hobson and Kardynal 2016). Analysis of multiple isotope ratios could provide information to corroborate the presence of multiple migratory populations or parts of populations using different migratory strategies (Bridge et al. 2014).

In metabolically inert tissues, such as keratin‐based feathers and claws, isotopes are only incorporated during the period of growth and then remain stable over time and so will reflect the diet of the individual during their period of growth (Wassenaar 2019). In a species which completes a pre‐migration moult on the breeding ground, feather isotope values would reflect conditions on the breeding ground, while feathers grown during migration or at wintering sites would have distinct isotope signatures reflecting those different geographic locations. Claws, in contrast, are continuously growing and provide a continuous record over a period of several weeks to months (Bearhop et al. 2003), potentially including values from both wintering grounds (from claw tips) and breeding grounds (from base of claws). However, there have been few studies of claw growth rates in passerines, so it is not clear how long a signature of the wintering ground is retained in this tissue type (Bearhop et al. 2003; Fraser et al. 2008). Analysing stable isotope ratios in both tissue types can therefore provide information that can be used to help differentiate between wintering, breeding, or moulting locations of migratory species (Hobson and Wassenaar 2019).

**FIGURE 1:**
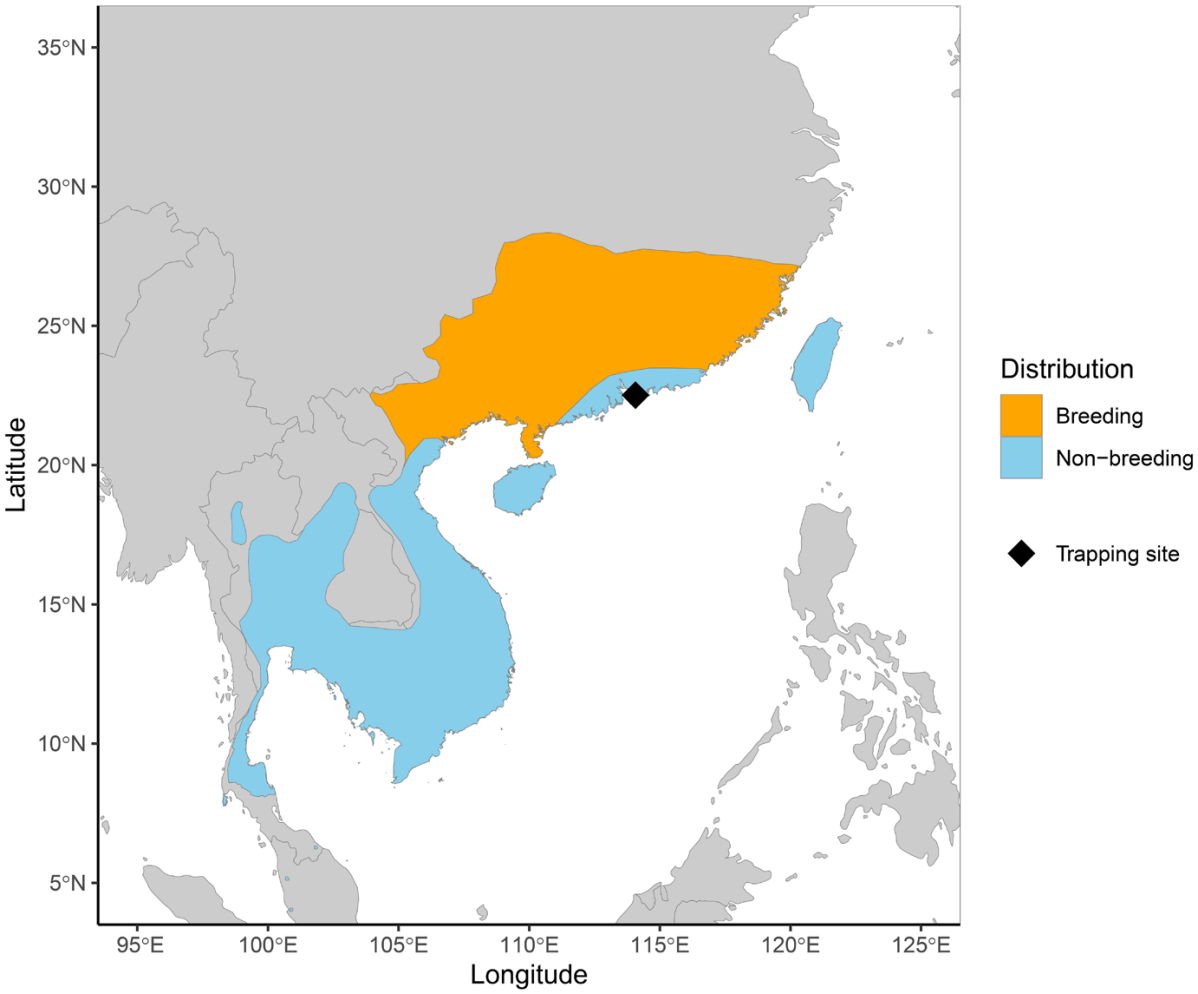
Global distribution of white‐shouldered starlings based on data from BirdLife International and Handbook of the Birds of the World (2024). Trapping location at the breeding site in Hong Kong indicated with a diamond on the map. Although these data show the species as present in Hong Kong only during the non‐breeding season, the species is known to breed in the area and can be found in Hong Kong throughout the year.

White‐shouldered starlings ( *Sturnia sinensis* ) are mid‐sized cavity‐nesting passerines that breed in southern China and northern Vietnam (Figure 1). Although the species is listed as Least Concern on the IUCN Red List and the global population is currently thought to be stable (BirdLife International 2023), population declines were previously reported in some parts of its range, including Hong Kong (Carey et al. 2001). Although considered common in Hong Kong in the first half of the twentieth century (Herklots 1953; Vaughan and Jones 1913), the local breeding population showed a decline in numbers in the late 20th century, with an estimated population of fewer than 50 pairs in the late 1990s (Carey et al. 2001). Since then, the population has increased in abundance, driven partly by the use of artificial nest sites, including nest boxes erected specifically to encourage breeding (Pang et al. 2023).

The white‐shouldered starling is described as a full or partial migrant, with at least some populations migrating to wintering grounds in Southeast Asia, coastal China, and Taiwan (Craig and Feare 2020). The species has been recorded as an occasional vagrant to Borneo, the Philippines, Singapore, Korea, and Japan (Craig and Feare 2020). According to the literature, populations in Hong Kong and Northern Vietnam are resident (Craig and Feare 2020). White‐shouldered starlings are present in Hong Kong throughout the year, with increased numbers present during spring and autumn migration periods (Carey et al. 2001), but observations from local ringers suggest that the Hong Kong population is at least partially migratory (P. J. Leader, pers. comm.). Individuals previously colour‐banded in Hong Kong in the winter were never re‐sighted in the summer, nor were breeding birds spotted in the winter. In contrast, birds banded in the summer returned to the breeding site in subsequent years, often returning to the same location where they had been banded (P. J. Leader, unpublished data).

Using light‐level geolocators and stable isotope analyses, we aimed to determine whether the breeding population of white‐shouldered starlings in Hong Kong is resident or fully or partially migratory, with either the whole breeding population leaving Hong Kong to winter elsewhere or with some proportion of the population being migratory or resident. The use of both geolocator and isotope data further allowed us to explore the consistency between results derived from both marker types, information that will help determine the utility of using SIA for further studies of migratory movements throughout the region.

## 2 Methods

### 2.1 Study Species, Study Site, and Trapping Method

White‐shouldered starlings breed at several sites throughout Hong Kong. We conducted our study at the Mass Transit Railway Corporation (MTRC) Wetland Enhancement Area at Lok Ma Chau (ca. 22.510° S, 114.06° E), where nest boxes have been provided to encourage species recovery. Breeding birds are typically present on site from March until August, with previous observations of birds banded as nestlings suggesting high site fidelity (P. J. Leader, unpublished data). Site fidelity is important in geolocator studies, as individuals must be recaptured to retrieve the data collected from the geolocator. We trapped adult birds in nestboxes using nestbox traps following ethical trapping under permits issued by the Hong Kong Government (AFCD Permit #AF FR CON 09/50 Pt. 34), Hong Kong Department of Health (Permit # (21‐154) and DH/HT&A/8/2/3 Pt. 22) and The University of Hong Kong's ethics authority (CULATR Protocol #5643‐21).

### 2.2 Sample Collection and Geolocator Fitting

We fitted each bird with a numbered aluminium leg ring so that individuals could be identified upon recapture. Individuals were sexed based on plumage differences, especially wing pattern (males show more extensive white in the lesser and median wing coverts) and body plumage (males are light grey, often with white crown and throat, whereas females are browner in tone; Pang et al. 2023).

In 2021, we fitted 20 adult birds (10 males, 10 females) with a 0.72 g light‐level geolocator (Intigeo‐P65B1‐7‐NOT; Migrate Technology, UK). We attached geolocators using a leg harness adapted from the method proposed by Rappole and Tipton (1991). We created the harnesses before catching the birds by threading the geolocator onto a length of elastic jewellery cord with a diameter of 0.8 mm and a length of 18–20 cm to create two loops. After capture, we fitted these loops around the legs of the bird and adjusted the length to fit the individual, tied the loose ends of the cord together, trimmed excess cord (about 10 cm of excess cord removed for each harness) and glued the ends of the cord to the tag using superglue to prevent fraying. This attachment technique is widely used on passerines and has been found to have no noticeable impacts on behaviour (Bell et al. 2017). Mass of the geolocators with harnesses ranged from 1.5% to 2.0% of individuals' body mass (average 1.8%). In the breeding season of 2022, we re‐trapped birds at the study site to recover the geolocators, which we removed from recaptured birds. For each re‐trapped bird that had been fitted with a geolocator, we collected a new feather sample and a claw sample, after which the birds were released.

### 2.3 Movement Analysis

We recovered 8 of the 20 deployed geolocators and offloaded data from these geolocators and corrected for time drift in the Intigeo interface (Migrate Technologies, Cambridge, UK). One data logger was returned to the manufacturer for data offloading because it had stopped recording prior to retrieval; subsequent review of the data shows that the logger failed on 4 May 2022 after the bird had returned to Hong Kong, and the complete cycle of migration had been recorded, although it was not possible to correct for time drift on this logger.

We estimated locations based on recommended methods described in Lisovski et al. (2019). Using the drift‐corrected light‐level recordings, we estimated twilight times using the GeoLight package (Lisovski et al. 2023) in R, using a light‐level threshold of 2.5 for the estimation of twilight times, as this was above the night‐time light level throughout the year. We corrected a few estimated twilight times that obviously differed visually from neighbouring twilights, but we were conservative with twilight corrections. Because light estimation can be affected by individual behaviour and differences between data loggers, we calibrated twilight estimations for each tag using the GeoLight package (Lisovski et al. 2023). We used calibration dates during a period when birds were expected to be at the breeding site or nearby area, involving all days from the date of tagging until 19 July 2021 (the last date of breeding activity at the site in that year) and from 4 April 2022 until the date of recapture. Large post‐breeding flocks of adult and juvenile *S. sinensis* usually remain in the surrounding area through July and August, so we expected that all tagged birds would remain close to the breeding site throughout this period but were unsure of the exact departure date. We based spring calibration on an arrival time 2 weeks before the peak egg‐laying date of the first brood, allowing time for birds to form pairs and build nests before egg laying started. We used a gamma distribution to estimate the zenith angle and error distribution for the estimated twilight light level of each individual tag, which were then used in later location estimates.

Using these estimated twilight times and zenith angles, we calculated preliminary location estimates for all dates through the year using the package GeoLocTools (Lisovski et al. 2019). We refined these locations using the package SGAT (Wotherspoon et al. 2016). After developing an initial estimate of locations based on the twilight times and sun zenith angles for the entire year, we confined the locations until 19 July 2021 and after 4 April 2022 to the breeding site location. We grouped locations with a similar location into stopovers based on the similarity of twilight timing: locations with an estimated movement of less than 200 km over a period of 5 days were grouped into a single stopover location. We used a relatively small distance threshold between estimated locations (200 km) to account for the possibility that the migration route could involve short hops along the coastline or short‐distance changes in location within the wintering area. Finally, we estimated the true location of these stopover sites using a multi‐chain Monte Carlo process to estimate the most likely location of the wintering site and any stopover locations. We estimated the expected distribution in a box bounded by the coordinates 0°–35° North and 105°–130° East, using a mask of land distribution within this region to limit estimated stopovers to terrestrial locations. Because of large variation in estimated locations (especially latitude) during the equinox periods, we excluded data within 15 days on each side of the equinox (8 September−8 October 2021 and 5 March−4 April 2022) from the location estimates; this limited our ability to confirm migratory routes between breeding and wintering sites because most birds migrated during these equinox periods.

Because migration dates coincided with the equinox periods, location estimates during migration were poor. This error mostly affected the latitudinal estimates, rather than longitude, and this population shows a strong east–west component in the migration route. To try to refine the estimated dates of migration, we looked for abrupt changes in longitude of the initial location estimates (defined as a date when the longitude changed by > 1° and did not return the following day) that we reasoned would reveal exact dates of departure or arrival at breeding and wintering sites.

### 2.4 Stable Isotope Analyses

We conducted SIA to measure isotope ratios in feathers and claws collected from birds fitted with geolocators. In 2001, we collected two tail feathers from each bird fitted with a geolocator. Feathers were plucked and stored in envelopes. For each bird with a geolocator that we re‐trapped in 2022, we again collected 2 tail feathers and additionally collected a short section (1–2 mm) of the middle claw on each foot on a piece of tape using a pair of small metal scissors. Claws were stored in the same envelopes as the feather samples. We collected feather samples on the same day that geolocators were attached in 2021 and collected both feathers and claws on the same day that the geolocator was retrieved from each bird in 2022. Feathers collected from birds in 2022 were presumed to have been grown either in Hong Kong at the end of the 2021 breeding season or on the wintering ground for the 2021–2022 breeding season, depending on the moult pattern in this species. Feathers collected in 2021 were analysed for comparison to feathers collected in 2022 to test for consistency between years, with the assumption that this would suggest that the bird had returned to the same breeding location in both years.

To prepare feathers for SIA, we soaked feathers in a 2:1 dichloroform: methanol solution for 12 h to remove surface oil and dirt (Wassenaar 2008). After 12 h, the feathers were rinsed with distilled water twice before being dried in an oven at 60°C to remove any remaining organic solvent (Hobson 1999). After cleaning, we cut sections from the cleaned feathers, cut the sections into small pieces, and then weighed them. We weighed feathers for H analysis to ~0.35 mg before placing them into a 5 × 9 mm silver capsule. Feathers for CNS analysis were weighted to ~0.1 mg and placed into tin capsules for analysis. Claw samples were only used for the analysis of δ^2^H. We first washed claw samples with acetone to remove any dirt or tape residue remaining on the claws, followed by a thorough rinsing with distilled water to remove the solvent. We then weighed the claws to ~3 mg and placed them whole into capsules for analysis. As the exchangeable hydrogen in the samples can easily exchange with the hydrogen in laboratory air (Wassenaar and Hobson 2003), samples and standards used for H/O analysis were allowed to equilibrate with laboratory air for a week before analysis.

The samples and the equilibrated keratin standards were analysed on an Elementar Pryo cube EA/Isoprime Precision IRMS (HO analysis) and on a Thermo EA‐Isolink‐Delta V Advantage IRMS (CNS) in the Stable Isotope Mass Ratio Spectrometry (SIMRS) Laboratory at the University of Hong Kong. Standards used include Kudu Horn Standard (KHS) and Caribou Hoof Standard (CB) provided by USGS, and benzoic acid (Merck). Finally, we adjusted hydrogen and oxygen isotope ratios of feather samples, claw samples, and the keratin standards using the standard VSMOW (Vienna Standard Mean Ocean Water). For the CNS isotopes, samples were adjusted using IAEA‐S1 and IAEA‐S2 (silver sulfide) standards.

## 3 Results

### 3.1 Geolocator Retrieval

Of the 20 individuals fitted with geolocators, we recaptured 12 in 2022 (five males and seven females), 8 of which (three males and five females) had their geolocators intact. Geolocators were missing on four of the recaptured individuals, most likely because their harnesses had snapped, as none of these individuals had the jewellery cord still attached. None of the recaptured birds showed any injury or feather loss due to geolocator attachment. We were able to retrieve data from all eight of the recovered geolocators, although one had stopped recording after returning to the breeding area before recapture.

#### 3.1.1 Movement Analysis From Geolocators

**FIGURE 2:**
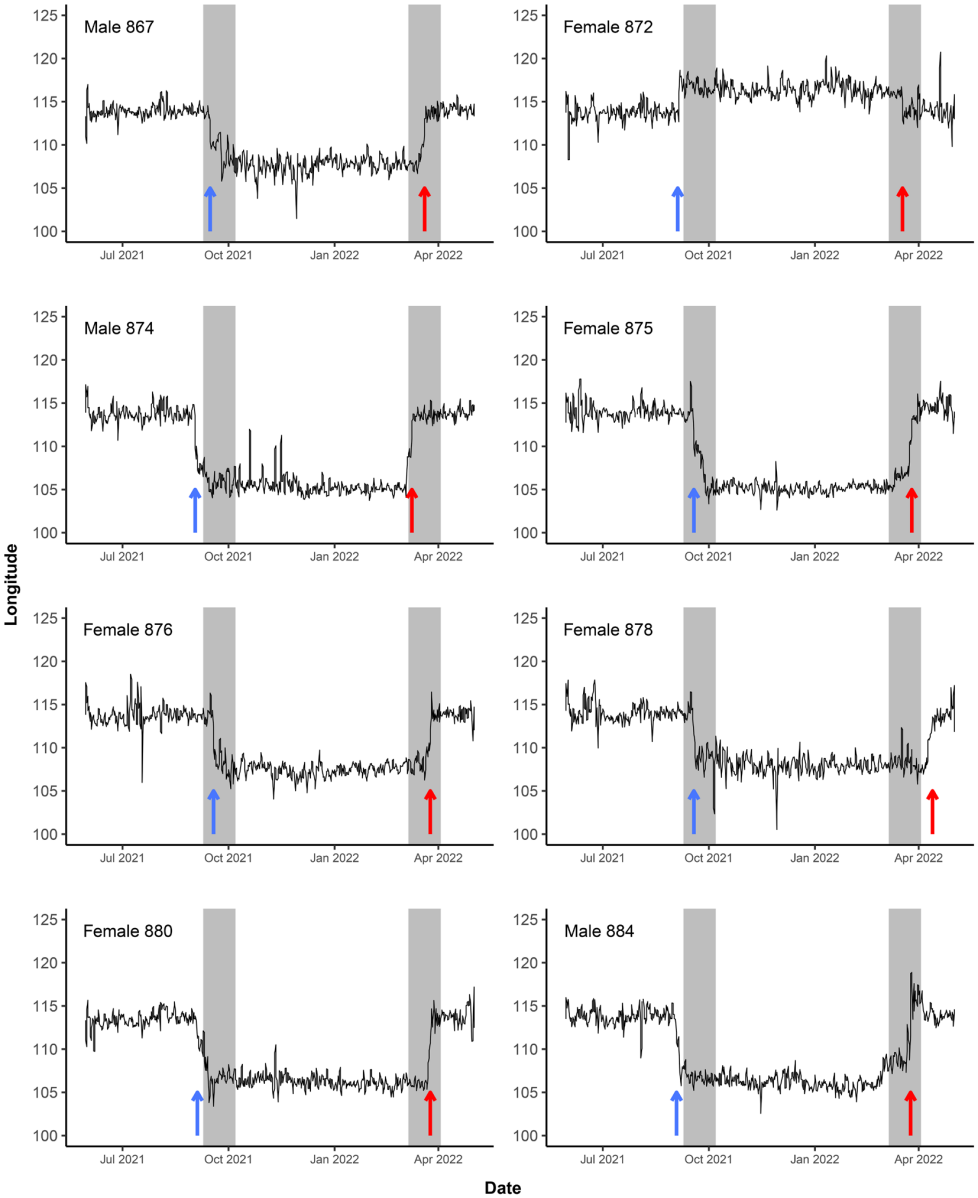
Estimated longitudinal location of the eight tracked *white‐shouldered starlings* based on daily twilight times. Periods within 2 weeks on each side of the equinox are shaded in grey. Arrows highlight the estimated dates of departure from Hong Kong (blue) and arrival in Hong Kong (red).

**FIGURE 3:**
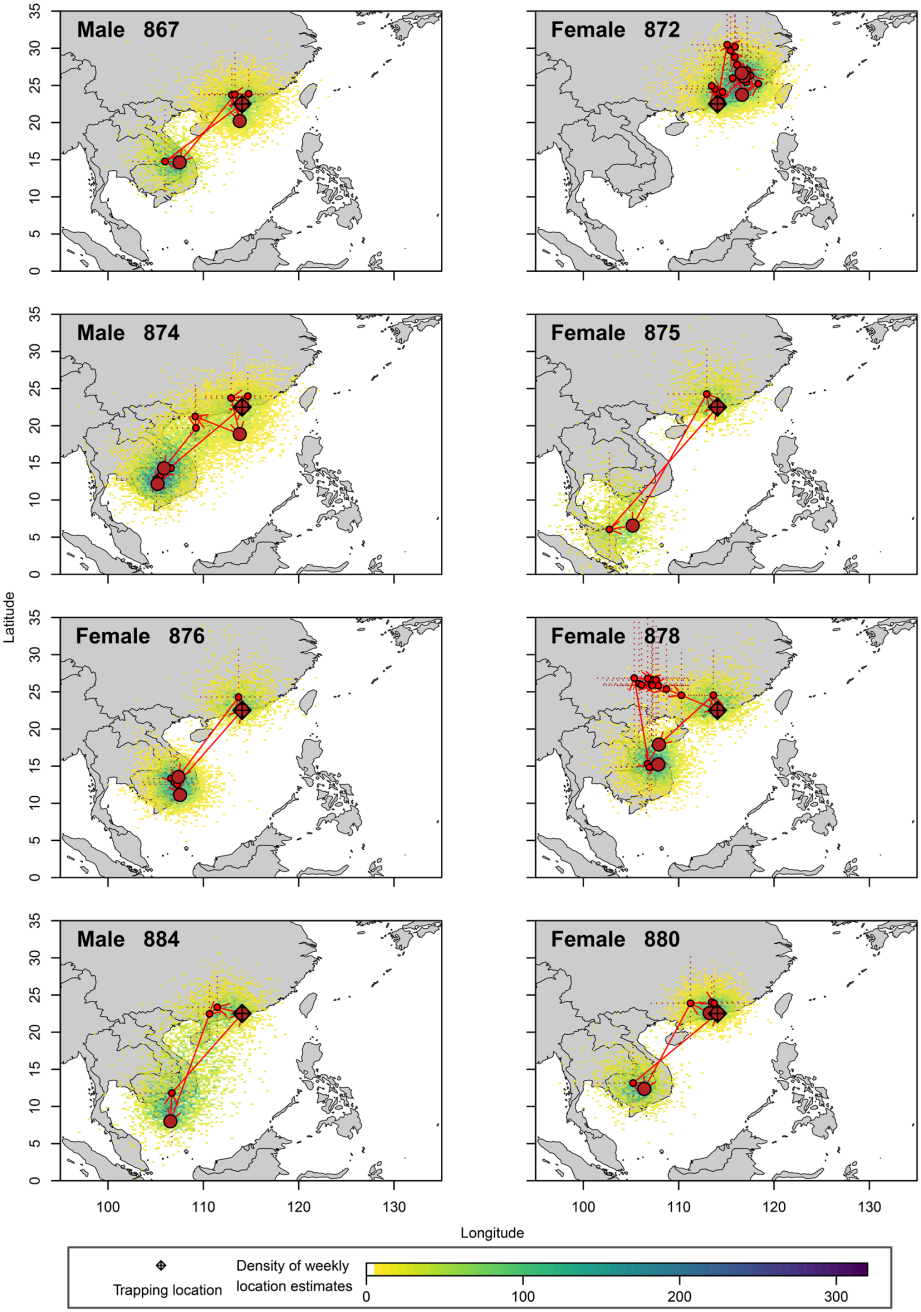
Density of estimated locations of white‐shouldered starlings (blue = high density or estimated locations, yellow = low density), with median estimates of stopover locations (large dark red circles = stopovers lasting multiple days, small red circles = short migration stopovers of less than 1 day, dotted lines = 95% confidence intervals) and routes between successive locations.

Analysis of data retrieved from geolocators revealed that 7 of the 8 birds from which we retrieved geolocators overwintered in southern Vietnam, southern Laos, or Cambodia where they were present from October 2021 until March 2022 (Figures 2 and 3). Estimated mid‐winter locations in this region were on average 1470 ± 347 km (range 1040–2013 km) from the study site in Hong Kong (Table 1). This area includes wetlands around the delta of the Mekong River and the floodplain of the Tonle Sap. In contrast to the other individuals, Female 872 followed a very different migration strategy in 2021, moving slightly east or northeast of Hong Kong to overwinter in eastern Guangdong, southern Fujian, or southern Jiangxi province in China (Figure 3), approximately 527 km from the study site. Several birds (872, 874, 876, 878) were estimated to change location during the winter.

**TABLE 1:** Estimated departure dates from the breeding site (Depart date 2021), arrival at the breeding site (Arrive date 2022), and dates at various points during the migration period (Location date).

No.	Sex	Depart date (2021)	Arrive date (2022)	Location date	Long.	Lat.	Dist. from HK (km)
867	M	15 Sept	20 Mar	^a^ 17 July (au) (sp)	114.07 113.81 107.51 114.07	22.51 20.22 14.63 22.51	0 256 1116 0
872	F	4 Sept	18 Mar	^a^ 31 July (au) 3 Nov 16 Dec (sp)	114.07 114.17 116.84 116.65 116.61 114.07	22.51 22.65 26.23 23.74 26.65 22.51	0 19 500 297 527 0
874	M	2 Sept	9 Mar	^a^ 17 July 4 Sept 28 Nov (sp)	114.07 113.82 105.87 105.20 114.07	22.51 18.87 14.28 12.17 22.51	0 406 1258 1484 0
875	F	18 Sept	24–26 Mar	^a^ (au) (sp)	114.07 105.18 114.07	22.51 6.56 22.51	0 2013 0
876	F	18 Sept	24–25 Mar	^a^ (au) 31 Dec (sp)	114.07 107.38 107.55 114.07	22.51 13.54 11.12 22.51	0 1222 1443 0
878	F	18–19 Sept	9–13 Apr	^a^ (au) 31 Dec 09 Apr	114.07 107.93 107.86 114.07	22.51 17.91 15.22 22.51	0 819 1040 0
880	F	4–10 Sept	25 Mar	^a^ 27 July (au) (sp)	114.07 113.24 106.40 114.07	22.51 22.56 12.36 22.51	0 84 1389 0
884	M	3–6 Sept	2–25 Mar	^a^ 06 Sept (sp)	114.07 106.54 114.07	22.51 8.00 22.51	0 1803 0

*Note:* Locations were estimated based on recommended methods described in Lisovski et al. (2019), with the estimated date of arrival at that particular location and the distance of the location from the breeding site in Hong Kong. Sex: M = male, F = female. Long. = longitude, Lat. = latitude, Dist. from HK is the estimated distance from the breeding site at the data capture date.

^a^ The date of tagging at the breeding site, (au): Migration occurred during the autumn equinox period (9 September–7 October 2021), (sp) migration occurred during the spring equinox period (6 March–3 April 2022).

Most migratory movements coincided with the equinox, and migration dates could not be estimated by the movement model. However, as the direction of movement was largely in an east–west direction, it was possible to estimate the dates of migration based on initial estimates of longitude, which are more reliable than latitudinal estimates from geolocator data, especially close to the equinox. Based on longitudinal data from the geolocators, all birds departed from the breeding area between 02 and 18 September 2021 and returned between 09 March and 13 April 2022 (Table 1, Figure 2). During both the autumn and spring migration seasons, some birds appear to have travelled between wintering and breeding grounds in 1 day, while others (874, 878, 880, 884) stopped for a day or two during migration (Figure 3).

For four individuals (867, 872, 874, and 880), the movement analysis estimated movements away from the breeding area in mid‐July. However, for two of these birds (867, 874), the estimated locations were at unsuitable offshore locations to the south of Hong Kong. These locations may be the result of erroneous latitudinal calculations, perhaps resulting from a difference in the behaviour of the birds (e.g., if they spent more time within tree canopies at this time) or weather conditions (cloudy conditions associated with rainfall during the Hong Kong wet season may influence the estimated twilight time).

### 3.2 SIA

Our SIA included samples from 11 individuals—the eight individuals for which we retrieved geolocators in 2022, and an additional three individuals that were fitted with geolocators that were lost before recapture. We did not obtain results for H/O from two individuals in 2021 and for one individual in 2022 (Appendix S1). There were no significant differences in mean isotope ratio values between years for any of the five elements (HOCNS) analysed (Table 2).

**TABLE 2:** Mean ± SD isotope ratio values for each of the five elements analysed in feathers collected in 2021 and 2022, and claws in 2022.

	Feathers		Claws	
	2021	2022	2021 vs. 2022	2022
δ^2^H	−58.29 ± 6.55	−58.97 ± 4.92	*p* = 0.80	−51.74 ± 4.01
δ^18^O	11.52 ± 1.48	10.5 ± 1.4	*p* = 0.13	12.81 ± 1.06
δ^13^C	−24.26 ± 0.40	−24.45 ± 0.59	*p* = 0.41	—
δ^14^N	12.58 ± 0.70	11.9 ± 1.13	*p* = 0.12	—
δ^34^S	−8.27 ± 4.96	−6.97 ± 4.46	*p* = 0.54	—

*Note:* *p* values from two‐tailed *t*‐tests comparing mean values from feathers collected in 2021 and 2022.

**FIGURE 4:**
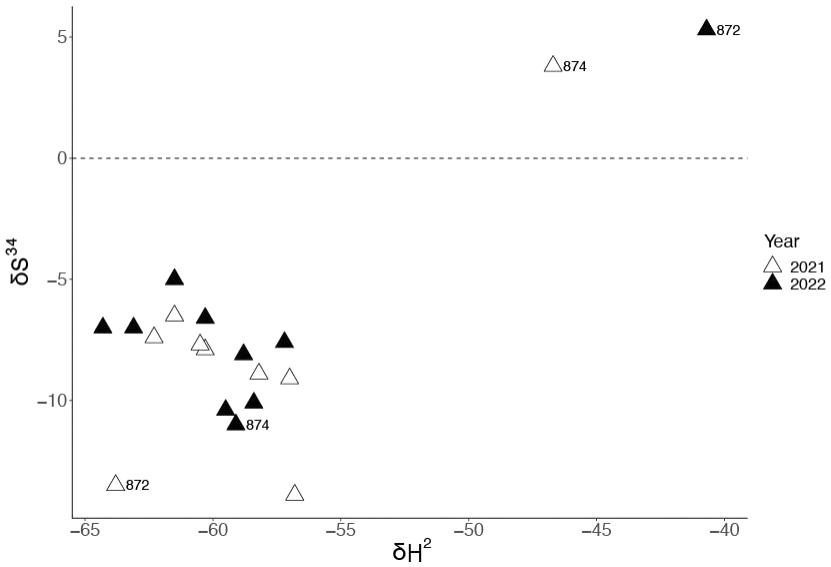
Stable isotope analysis from feathers collected during the breeding season in 2021 (prior to placing geolocators) and feathers and claws collected during the breeding season in 2022 (after retrieving geolocators). Data for female 872 and male 874, which differ between the 2 years, are labelled.

The values for δ^2^H and δ^34^S from the feather collected in 2022 from Female 872, who migrated NE from Hong Kong over the 2021–2022 winter according to the geolocator data, differed from those individuals that migrated SW from Hong Kong (Figure 4; Appendix S1). δ^2^H for this individual differed by 2.5 standard deviations from the mean for δ^2^H (*Z*‐score; *Z* = 2.49), close to the maximum for this sample size. In contrast, in 2021, the feather isotope ratios from this individual did not differ from the other individuals (Figure 4). Isotope analysis of the feathers collected in 2021 identified a different individual (Male 874) that had δ^2^H and δ^34^S values that differed from the other individuals (δ^2^H *Z*‐score; *Z* = 2.63) and showed a pattern consistent with Female 872, who had migrated NE to a wintering site in the following year. Both isotope and geolocator data indicate that this individual migrated to SE Asia along with the other birds in the winter of 2021–2022 (Figure 4).

The isotope values measured in claws were more variable than for feathers, but no clear outliers were observed (Appendices S1 and S2). Claws were only collected in 2022, so no comparison between years was possible.

## 4 Discussion

Here, we used a combination of extrinsic and intrinsic markers to provide information on the migratory pathways used by *white‐shouldered starlings* breeding in Hong Kong. Our data provide insights into the migration routes of this species and evidence that while white‐shouldered starlings are present year‐round in Hong Kong, the wintering and breeding populations likely constitute birds of different origins. Intriguingly, one bird migrated to a different breeding site in that same year as indicated by both geolocator and isotope data, and another individual is inferred to have switched wintering locations between years based on isotope data alone. Isotope data support results from geolocator data, providing additional evidence that SIA can provide insights into migration patterns in Asian songbirds. These results highlight the benefits of combining both geolocator and SIA to identify migratory patterns for birds along the EAAF.

Our finding that the main wintering sites for the Hong Kong population of this species are in SE Asia is consistent with published information on known wintering grounds for this species (Fink et al. 2022). Based on modelling from data submitted to eBird from 2007 to 2021, it is estimated that between 30% and 45% of the global population of *white‐shouldered starlings* winters in southern Vietnam and Cambodia between November and March, whereas only around 1% of the population winters in eastern Guangdong and western Jiangxi (Fink et al. 2022). Geolocator data from our study suggest that some individuals moved between breeding and wintering sites in a single day, travelling more than 1000 km between the two sites. Other Asian starling species have also been shown to be capable of such long‐distance flights during migration. For instance, chestnut‐cheeked starlings ( *Agropsar philippensis* ) migrate between breeding sites in Japan and wintering sites in SE Asia. While chestnut‐cheeked starlings appear to island hop as they move between the two sites, some individuals also undertake flights of more than 1000 km in some parts of their migratory routes (Koike et al. 2016).

Although the Hong Kong *white‐shouldered starling* population has previously been recorded as being resident (Craig and Feare 2020), our data show that this species is at least partially migratory, possibly following a ‘chain migration strategy’ in which all populations migrate south to wintering sites (Alerstam and Hederström 1998; Newton 2008). Under this scenario, the Hong Kong breeding birds leaving the site to winter elsewhere (predominantly to wintering sites SW of Hong Kong) would be replaced in Hong Kong in winter by birds from further north. Despite our observation that all birds carrying geolocators left the breeding site at the end of the breeding season, it is still possible that some portion of the population is sedentary, with some individuals remaining to overwinter at or near the breeding site in Hong Kong. Indeed, the fact that one individual from our study (Female 872) migrated a short distance northeast (based on geolocator data) shows that some individuals remain in southeast China throughout the winter, giving a possibility that differing migration strategies coexist within the population. In addition, the combination of geolocator and isotope data suggest the possibility that individuals may switch wintering locations between years. Isotope ratios measured in the feathers collected in 2022 corroborated geolocator data, with Female 872 having markedly different δ^2^H and δ^34^S values than the rest of the individuals which migrated to wintering sites in SE Asia. This difference was not observed in feathers collected in 2021, suggesting that Female 872 wintered in with the other individuals in SE Asia in 2020–2021—and if true would indicate that this individual changed wintering sites between years.

Based on isotope data alone, we identified another individual (Male 874) which appears to have switched wintering sites between the 2 years. As we do not have geolocator data from the 2020–2021 migration period to confirm this observation, we interpret this pattern cautiously. Male 874 hatched at the breeding site in 2020, so the feather that we analysed could have been a retained juvenile tail feather or a feather replaced soon after fledging and thus not grown on the wintering ground. However, we tagged two other individuals (875 and 880) which were also born at the site in 2020, and the isotope ratios measured from their tail feathers, collected during the same breeding season, were similar to the other tagged birds in both 2021 and 2022, suggesting that these birds do not routinely retain juvenile tail feathers. This provides additional evidence to support the observation that Male 874 migrated to a different location in that winter. While we cannot determine whether Female 872 and Male 874 overwintered in Hong Kong or undertook short migrations to SE China based on the isotope data alone, our results highlight the potential utility of SIA to uncover migration patterns where wintering (or moulting) sites differ for birds migrating along the EAAF. Further research on expanded datasets could help to further explore the utility of a multi‐isotope approach and provide more insight into the variation of isotope ratios that could be used to develop this technique for applications throughout the flyway.

Our data do not allow us to test hypotheses for why white‐shouldered starlings might employ different migration strategies between individuals within the same population (partial migration) or within an individual between years. White‐shouldered starlings migrate in flocks, sometimes with other starling species (J. A. Allcock, pers. obs.), and it is possible that wintering site location is influenced by flock membership. Potentially, Female 872 and Male 874 joined a flock containing individuals that remained in south China through the winter rather than moving to Southeast Asia. Both partial migration and annual changes in migration strategy have been shown to be influenced by environmental or genetic variables (Schwabl 1983; Newton 2008; Rivalan et al. 2007; Ogonowski and Conway 2009; Ranck et al. 2023). Migration is costly—both in terms of mortality and reduced reproductive success (Newton 2008), so shorter migration routes, or the loss of migration behaviour altogether, may evolve when climate change leads to changes in wintering conditions at breeding sites that allow birds to remain overwinter (Rivalan et al. 2007). Unidirectional annual switches in migration strategy have been linked to age or social dominance (Schwabl 1983; Ogonowski and Conway 2009) while changing back and forth between years has been linked to changes in the environment (great bustard *Otis tarda* Palacín et al. 2011, American kestrels *Falco sparverius* Ranck et al. 2023).

Mixed migration and moulting strategies have been shown in other starling species (e.g., common starling *Sturnus vulgaris* , Vīgants et al. 2023). In one population of common starlings, half of the tracked individuals stayed at the breeding site until October, presumably completing their moult prior to migration, while the other half left the breeding site in mid‐June and completed their moult during their migratory period—potentially related to variability in climate throughout the region (Vīgants et al. 2023). The utility of SIA to provide usable information on migration patterns is highly dependent on knowledge of moult patterns in the species being studied. Little is known about the moult pattern in White‐shouldered Starlings, and so it is not known when the tail feathers in this species are moulted. Most starling species undertake a complete moult after breeding and before migration (Craig and Feare 1998); however, the timing of moult varies between species, with some species (including rosy starling *Pastor roseus* and spot‐winged starling *Saroglossa spilopterus*) undertaking a complete moult on the non‐breeding grounds, and some (e.g., violet‐backed starling *Cinnyricinclus leucogaster* , wattled starling *Creatophora cinerea* , and common starling *Sturnus vulgaris* ) showing regional variation in the timing of moult relative to migration (Traylor 1971; Craig and Feare 1998; Vīgants et al. 2023). During our study, adult female *white‐shouldered starlings* began primary moult before the end of the breeding season, but we have not observed body moult on this species at the study site. We conducted an informal review of photographs submitted to eBird (www.ebird.org) and found *white‐shouldered starlings* apparently in tail moult between 24 September and 26 October, as well as on 4 December and 21 January; this suggests that the tail feathers would be replaced shortly after arrival at the wintering grounds (although alternatively these may involve replacement of feathers due to damage). These results underscore the importance of collecting data on moult patterns in birds using this flyway.

In contrast to results from the isotope analysis on feathers, isotope ratios measured in the claw samples did not show Female 872 individual as an outlier. As the claws grow continuously, even during the migration period, claws can record isotopic ratios of diet over a period of up to 6 months (Bearhop et al. 2003). Therefore, a claw sample could contain variation in isotopic ratios along its length as the claws continuously grow as the bird moves between different sites during migration (Hobson and Wassenaar 2019). While this variation can provide useful information about changes in geographic location, it can confound results when whole claws are used in analyses, therefore limiting the utility of claws for estimating geographic position with any certainty (Mazerolle and Hobson 2005).

In conclusion, the results from this study provide strong evidence that the Hong Kong population of this species is at least partially migratory, with a predominant southeast‐directed winter migration, while also providing evidence of mixed migration strategies in this population. At least one individual was found to undertake a shorter migration to winter NE of the breeding site, and geolocator and isotope data combined suggest that at least two individuals switched their wintering grounds between years. The combination of both extrinsic and intrinsic markers provides complementary information on migration strategies—while the geolocator provided spatially explicit information on the direction of migration and the location of wintering sites, stable isotope data supported findings from geolocator data and provided additional data to support the observation of mixed migration strategies in each year. In areas with largely homogeneous isotope values, as in large parts of SE Asia and southern China (Appendix 3), isotope analysis alone will unlikely be able to provide high‐resolution spatial assignments to migratory populations, but could be used to determine where geolocator studies would provide the most information on migratory strategies (Hobson and Kardynal 2016) and to support results from geolocator studies to determine the origin of migrants (Choi et al. 2020). Clarification of the true migration strategy of white‐shouldered starlings may require further tracking of birds from elsewhere within the breeding range or birds wintering in Hong Kong, as well as tracking of individuals in multiple years.

## Author Contributions

**Caroline Dingle:** conceptualization (equal), data curation (equal), formal analysis (equal), funding acquisition (supporting), investigation (lead), methodology (equal), resources (equal), supervision (lead), writing – original draft (lead), writing – review and editing (equal). **John A. Allcock:** conceptualization (equal), data curation (equal), formal analysis (equal), investigation (equal), methodology (equal), writing – original draft (supporting), writing – review and editing (equal). **Pia M. C. Ricca:** data curation (supporting), formal analysis (supporting), investigation (supporting), writing – review and editing (equal). **Paul J. Leader:** conceptualization (supporting), resources (supporting), writing – review and editing (equal). **Chloe E. R. Hatten:** formal analysis (supporting), writing – review and editing (supporting). **Juha Merilä:** conceptualization (supporting), funding acquisition (lead), methodology (equal), writing – original draft (supporting), writing – review and editing (equal).

## Ethics Statement

We trapped adult white‐shouldered starlings at nest boxes using nest box traps following standard protocols with permits from the Agriculture, Fisheries and Conservation Department (Permit # (146) in AF GR CON 09/51 Pt 7), the Hong Kong Department of Health (Licence to Conduct Experiments (21‐154) in DH/HT&A/8/2/3 Pt. 22), and the University of Hong Kong ethics authority (CULATR 5643‐21).

## Conflicts of Interest

The authors declare no conflicts of interest.

## Supporting information


Appendix S1.
Appendix S2.
Appendix S3.

## Data Availability

Analyses reported in this article can be reproduced using the data provided by CD and JAA. The raw geolocation data is deposited at www.movebank.org, and stable isotope data is provided in Appendices S1 and S2 of this paper.
