# Supplementary material for: Geolocator Tracking and Stable Isotope Analysis Suggest Mixed Migration Strategies in White‐Shouldered Starlings (Sturnia sinensis)
Source: Ecol Evol. 2025 Mar 30;15(4):e71151. doi: 10.1002/ece3.71151 (PMC11955405; doi:10.1002/ece3.71151)
Supplement: Supplementary file 1 — Appendix S1. Appendix S2. Appendix S3. [file ECE3-15-e71151-s001.docx]

**Appendix I.** Stable isotope ratios for 11 White-shouldered Starling *Sturnia sinensis* individuals for hydrogen (H), oxygen (O), and sulphur (S), measured in claws and feathers. All 11 individuals carried geolocators, but only 8 of these were recovered (indicated with an asterisk). Bold text indicates birds in each year which appear to have migrated to different wintering locations between years.

|  |  |  | **Feather 2021** | | | **Feather 2022** | | | **Claw 2022** | |
| --- | --- | --- | --- | --- | --- | --- | --- | --- | --- | --- |
| **Geolocator** | **Ring #** | **Sex** | **δ^2^H** | **δ^18^O** | **δ^34^S** | **δ^2^H** | **δ^18^O** | **δ^34^S** | **δ^2^H** | **δ^18^O** |
| **Tag** |  |  |  |  |  |  |  |  |  |  |
| 878 | TC02502 | F | - | - | -11.6 | -64.3 | 11.3 | -7.0 | -48.2 | 13.9 |
| **872** | **TC05106** | **F** | -63.8 | 9.3 | -13.5 | **-40.7** | **15.5** | **5.3** | **-53.8** | **13.2** |
| 871 | TC05115 | F | -57 | 10.5 | -9.1 | -57.2 | 10.5 | -7.6 | - | - |
| 866 | TC05126 | F | -61.5 | 11.2 | -6.5 | -58.8 | 11.9 | -8.1 | -58.7 | 12.8 |
| 881 | TC05181 | M | -58.2 | 11.4 | -8.9 | -59.5 | 11.2 | -10.4 | -48.8 | 12.1 |
| 875 | TC05578 | F | -62.3 | 10.2 | -7.4 | -61.5 | 11.7 | -5.0 | -52.2 | 13.1 |
| 880 | TC05585 | F | -60.3 | 9.2 | -7.9 | -58.4 | 10.6 | -10.1 | -55.3 | 12.1 |
| 876 | TC06325 | F | - | - | - | -60.3 | 11.3 | -6.6 | -52.3 | 10.9 |
| 867 | TC06355 | M | -60.5 | 12.1 | -7.7 | - | - | -9.2 | - | - |
| **874** | **TC05505** | **M** | **-46.7** | **10.3** | **3.8** | **-59.1** | **10.7** | **-11.0** | **-51.1** | **14.5** |
| 884 | TC06373 | M | -56.8 | 12.7 | -13.9 | -63.1 | 10.5 | -7.0 | -45.3 | 12.7 |


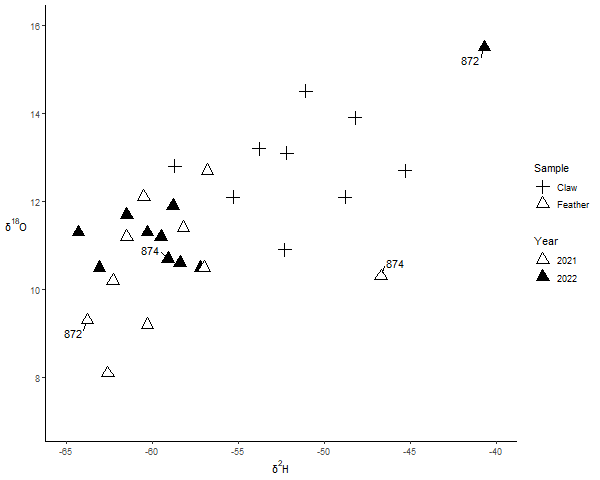


**Appendix 2**: Biplot showing *δ*^2^H and *δ*^18^O isotopic ratios from feathers (triangles) and claws (+).


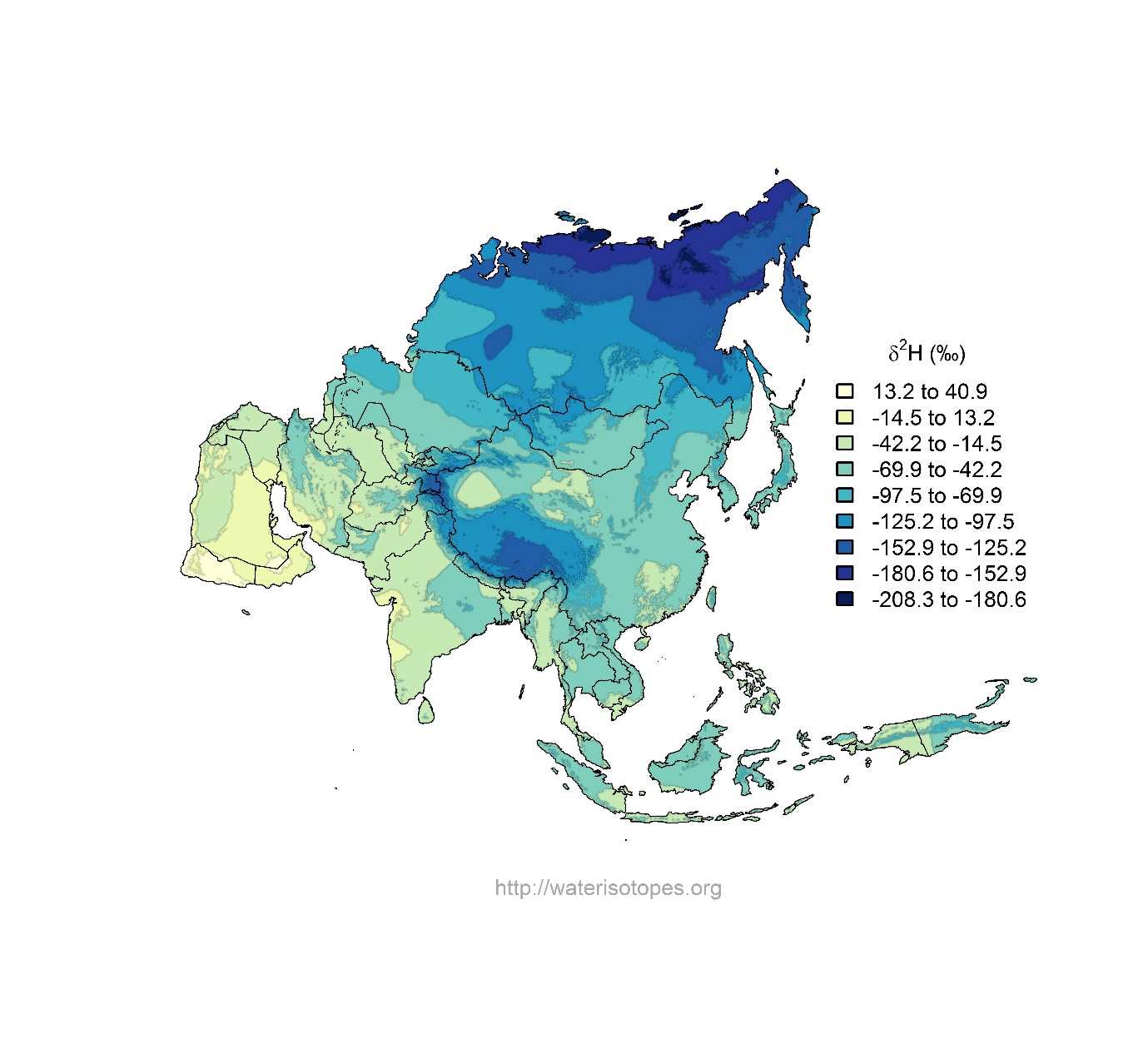


**Appendix 3**: *δ*^2^H isoscape in Asia based on OIPC 3.0 estimates of annual average precipitation. Figure downloaded from waterisotopes.org.
